# Study on Biofilm Formation Among Enterococcus Isolates and Association With Their Antibiotic Resistance Patterns

**DOI:** 10.7759/cureus.53594

**Published:** 2024-02-05

**Authors:** Pooja Nair, Sathish Sankar, P Neelusree

**Affiliations:** 1 Department of Microbiology, Saveetha Medical College and Hospitals, Saveetha Institute of Medical and Technical Sciences, Saveetha University, Chennai, IND; 2 Department of Microbiology, Saveetha Dental College and Hospitals, Saveetha Institute of Medical and Technical Sciences, Saveetha University, Chennai, IND

**Keywords:** medical, dental, well-being, disease, health, enterococcus gallinarum, enterococcus faecium, enterococcus faecalis, biofilm, antibiotic resistance

## Abstract

Background

Enterococci are a part of the normal intestinal flora of humans. They have emerged as one of the leading causes of nosocomial infection. The evolved antibiotic resistance mechanisms coupled with the virulence properties of enterococci have made it a successful pathogen.

Aim

This study aimed to determine the ability of biofilm formation among the clinical enterococci isolates and the antimicrobial resistance pattern of the strains.

Materials and methods

Clinical samples of patients who attended Saveetha Medical College and Hospital, Chennai, India, over six months. Identification and characterization of *Enterococcus *species were done using various biochemical tests. Antibiotic susceptibility patterns for each isolate were performed using the Kirby- Bauer disc diffusion method.

Results

The formation of biofilm formation was detected using the microtiter plate method. In total, 90 *Enterococcus *species were isolated; *Enterococcus faecalis *were 63 (70%)*, Enterococcus faecium *were 25 (28%) and* Enterococcus gallinarum *were 2 (2%)independently. *E. faecalis* displayed advanced resistance rates compared to other *Enterococcus *species. Resistance against penicillin was found in 42 strains (47%) and resistance to ampicillin was observed in 39 strains (43%). This was followed by resistance to high-level gentamicin in 35 strains (39%) and resistance to ciprofloxacin in 32 strains (36%). Resistance to vancomycin and linezolid also were noted in some strains.

Conclusion

Our results indicate that *E. faecalis* exhibits an increasing rate of antimicrobial resistance but lower biofilm conformation. The unique traits of *E. faecalis* raise concerns for the associated infections, especially hospital-acquired infections.

## Introduction

Enterococci are gram-positive, facultative anaerobic cocci that are present as commensals of the gastrointestinal system that have changed to a potential pathogen. Some enterococci are also found in the oral cavity and urogenital tracts [1]. They have emerged globally as one of the most important bacterial pathogens causing nosocomial infections due to their presence of virulence factors and rising antimicrobial resistance [2]. It is associated with bacteremia, urinary tract infections (UTIs), surgical site infections, tissue and wound infections, neonatal infections, and endocarditis. While some of the infections are community-acquired, others are nosocomial [2,3]. The risk factors associated with nosocomial infections are prolonged hospital stays, urinary catheters or any other in-dwelling devices, surgical procedures, care in intensive care units, serious concurrent underlying illness, organ transplant, multiple antibiotic therapies, and proximity to other patients with multidrug-resistant enterococci [4,5]. Enterococci species *Enterococcus faecalis,* *Enterococcus faecium,* *Enterococcus caseliflavus*, and *Enterococcus *​​​​​*hirae* as part of the commensal species, make up a significant component of the gut microbiota and account for most of the infections associated with humans [6]. Biofilm-producing *Enterococcus *species have increased virulence due to the presence of different factors including extracellular surface protein (ESP). The ESP is an adhesin protein that plays a major role in evading the host immune system.

Biofilm makes the enterococcal strains resistant to antibiotics. Biofilm-producing enterococci beget intermittent, habitual, and antibiotic-resistant infections. This biofilm conformation modulates the host immune system for increased action of virulence factors and antimicrobial action [7]. The biofilm allows the pathogen to avoid the phagocytic mechanism and helps in the exchange of virulence and antimicrobial resistance genes between the organisms [8,9]. In our study, we aimed to identify the biofilm-forming ability of the *Enterococcus *isolates obtained from clinical specimens including blood, respiratory specimens, pus, and wound aspirates of patients using microtiter plate assay to detect biofilm formation. This property was compared to the antimicrobial resistance pattern of the isolates.

## Materials and methods

Study design

The study is a prospective cross-sectional study with random sampling. Saveetha Medical College Institutional Ethical Committee issued approval SMC/IEC/2021/06/013. Patients reporting to the outpatient department of the hospital for various complaints were recruited. All the clinical samples that yielded positive for *Enterococcus *strains were selected for the study during the study period. Patients consented to participate in the study and those samples that were positive for *Enterococcus *were included. No additional sample was collected from the patients for this study. Patients who did not consent to participate and strains not confirmed for enterococci were excluded.

Period of study

The present study was conducted over a period of six months from January 2023 to June 2023. Clinical samples such as pus, tissue, urine, body fluids, and wound swabs were included.

Isolation and identification of *Enterococcus *species

Presumptive and confirmatory identification of the enterococci strains to species level was done by gram staining and using a battery of biochemical tests. The total sample size was 4954. *Enterococcus *species were isolated from 90 samples. All isolates were cultured in media based on established protocols. All the media, antibiotic discs, and chemicals were procured for the study from HiMedia Laboratories, Mumbai, India. The Bile Esculin test was used to confirm *Enterococcus *in bile by black color formation. Sugar assimilation tests using different sugars as the only carbon source were prepared for their growth detection and used for species differentiation based on their carbon assimilation profile.

Antibiotic susceptibility testing

An in vitro antimicrobial susceptibility test for all the enterococci was performed against the following antibiotics (HiMedia Laboratories, Mumbai, India) as per Clinical and Laboratory Standards Institute (CLSI) guidelines 2020 as follows: penicillin (10 mcg), ampicillin (10 mcg), ciprofloxacin (5mcg), vancomycin (30 mcg), teicoplanin (30 mcg), high-level gentamicin (120 mcg), erythromycin (30 mcg), linezolid (30 mcg), nitrofurantoin (30 mcg) and ceftriaxone (30 mcg) by employing Kirby-Bauer disk diffusion method. The zone of inhibition is measured in millimeters and compared against the CLSI standard for interpretation.

Screening for enterococcal biofilm production

The clinical isolates of enterococci strains were screened for biofilm formation using the microtiter plate method as per standard methods with some modifications. Freshly subcultured strains of the *Enterococcus *species in blood agar plates were inoculated in 1ml of tryptic soy broth (TSB) with 1% glucose and incubated overnight at 37°C. About 20 μl of this 24-hour-old bacterial growth and 180 μl of fresh TSB medium were added into 96 well polystyrene microtiter plates, matching to 0.5 McFarland turbidity standard. TSB medium (250 μl) alone in a well was taken as a negative control. All the isolates were inoculated in triplicates and incubated at 37°C for up to 24 hours. After incubation, the media from each well was decanted and rinsed twice with cold 1x phosphate-buffered saline (pH 7.4). The biofilm was fixed onto the surface by the addition of 150 μl methanol for 15 minutes and then washed and kept in an inverted position to air dry for 20 minutes. Then all the wells were stained using 0.1% crystal violet for 15 minutes. After incubation, the wells were rinsed under tap water, air dried, and destained by adding 150 μl of 33% glacial acetic acid and held for 20 minutes. The optical density (OD) value was read at 570nm. The steps were repeated for reproducibility in triplicates for each isolate and the mean value was taken. Based on the reports of the spectrophotometry read at 570 nm, the OD, and the cut-off OD (average of all the ODs of the negative control) were determined. The biofilm quantification and categorization were carried out based on the OD compared to the cut-off OD. Quantification of biofilm production was estimated for the isolated strains. The average of all the ODs of the negative control is considered the cut-off OD. The OD values were compared to the cut-off OD and classified as weak biofilm producer, moderate biofilm producer, and strong biofilm producer.

## Results

The number of *Enterococcus *isolated from a total of 4954 samples was 90, which accounts for 1.8% of the infection rate. Of the 90 enterococcal isolates, 53 (58.9%) were from urine samples, 16 (17.8%) from blood, 14 (15.5%) from wound swabs, four from tissue, and two from other body fluids. Among these, 50 (55%) were isolated from female patients and 40 (45%) from male patients of different clinical departments. From the above samples *E. faecalis* (n=63), *E. faecium* (n=25), and *Enterococcus gallinarum* (n=2) were isolated, respectively (Table 1).

**Table 1:** Clinical enterococci isolates identified from clinical samples

Clinical specimens	Total *Enterococcus *species	*Enterococcus faecalis*	Enterococcus faecium	Enterococcus gallinarum
Urine	53 (59%)	34 (64.15%)	17 (32.1%)	2 (3%)
Blood	16 (18%)	11 (68.7%)	5 (31.25%)	0
Tissue	4 (4%)	3 (75%)	1 (25%)	0
Swab	14 (16%)	12 (85.7%)	2 (14.3%)	0
Fluid	2 (2%)	2 (100%)	0	0
Others	1 (1%)	1 (100%)	0	0
Total	90	63 (70%)	25 (27.8%)	2 (2.2%)

**Figure 1:**
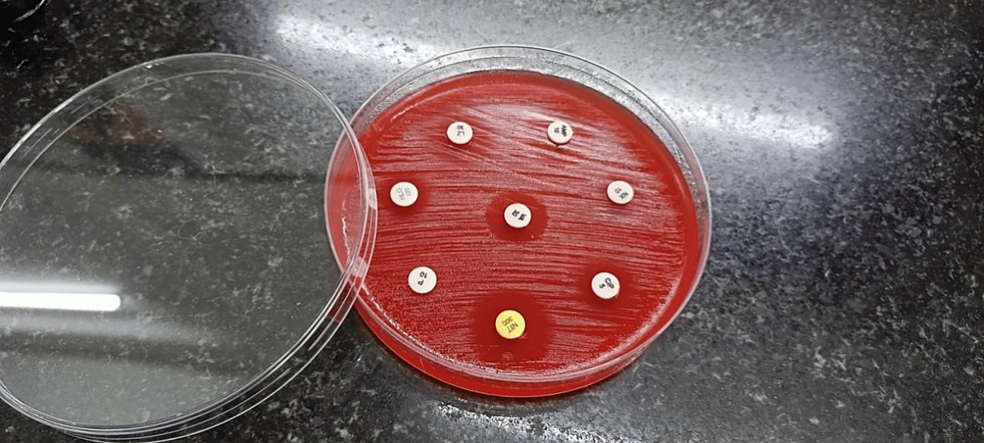
Antimicrobial susceptibility testing of the test isolates The *Enterococcus *species were tested for antimicrobial susceptibility with different antibiotics using the Kirby-Bauer method. The zone of inhibition is measured in millimeters and compared against the CLSI standard for interpretation.

The antibiotic study showed a markable susceptible pattern in blood agar using the disk diffusion method (Kirby-Bauer technique) (Figure 1). Among the 12 isolates that tested positive for strong biofilm production, seven were urinary isolates, two each from wound swabs and blood and one from tissue.

All the biofilm-producing enterococci isolates were resistant to penicillin, ampicillin, ciprofloxacin, and vancomycin. In these, two isolates were sensitive to nitrofurantoin, linezolid, and high-level gentamicin. Out of the two strong biofilm-producing wound swab isolates, one strain was sensitive to linezolid and vancomycin. Both were sensitive to high-level gentamicin. Among the two blood isolates producing strong biofilm, both were sensitive to linezolid and teicoplanin and resistant to vancomycin, erythromycin, penicillin, and ampicillin.

The identified strains were predominantly resistant to penicillin, ampicillin, and high-level gentamicin followed by ciprofloxacin and ceftriaxone. These isolates were least resistant to vancomycin, nitrofurantoin, and linezolid. The resistance pattern of the enterococcal species has been elucidated (Table 2).

**Table 2:** Antibiotic resistance pattern of enterococci

Antibiotics	Total *Enterococcus *species (n=90)	*Enterococcus faecalis *(n=63)	*Enterococcus faecium* (n=25)	*Enterococcus gallinarum *(n=2)
Ampicillin	39 (43%)	27 (43%)	11 (44%)	1
Ceftriaxone	29 (32%)	19 (30%)	10 (40%)	1
Ciprofloxacin	32 (36%)	22 (35%)	9 (36%)	1
Erythromycin	23 (26%)	16 (25%)	6 (24%)	1
High-level gentamicin	35 (39%)	25 (40%)	9 (36%)	1
Linezolid	8 (9%)	6 (10%)	2 (8%)	0
Nitrofurantoin	17 (19%)	12 (19%)	5 (20%)	0
Penicillin	42 (47%)	30 (48%)	11 (44%)	1
Teicoplanin	27 (30%)	20 (32%)	7 (28%)	0
Vancomycin	20 (22%)	14 (22%)	5 (20%)	1

**Figure 2:**
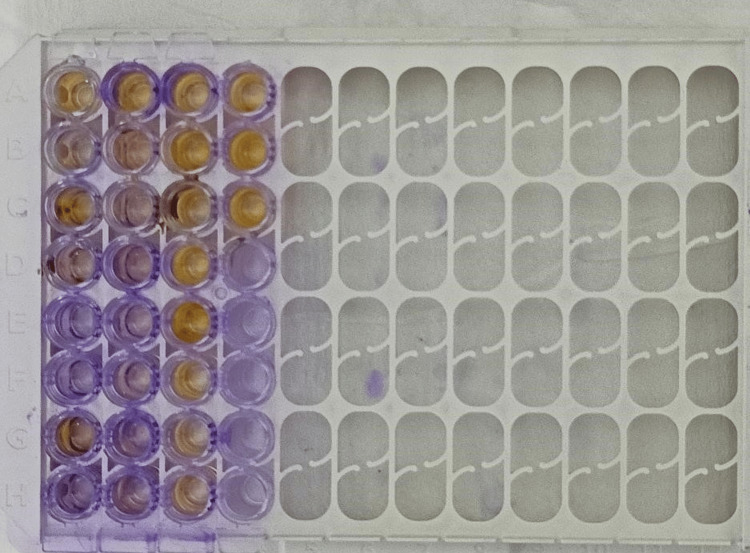
Estimation of biofilm production by the test isolates

Among the 90 isolates, 78 strains of enterococci showed biofilm formation and 12 strains did not show evidence of biofilm formation. Among the 78 biofilm-forming enterococci isolates, 12 isolates were strong biofilm producers (Figure 2).

*E. faecalis* isolates demonstrated increased biofilm formation than *E. faecium*. The extent of biofilm formation among enterococci isolates was identified in our study (Table 3).

**Table 3:** Number of different enterococci strains producing biofilms

Biofilm categorization	Enterococcus faecalis	Enterococcus faecium	Enterococcus gallinarum	Total
Strong biofilm producers	7	4	1	12 (13%)
Medium biofilm producers	41	17	0	58 (65%)
Weak biofilm producers	10	2	0	10 (11%)
Non-biofilm producers	5	2	1	10 (11%)
Total	63	25	2	

**Figure 3:**
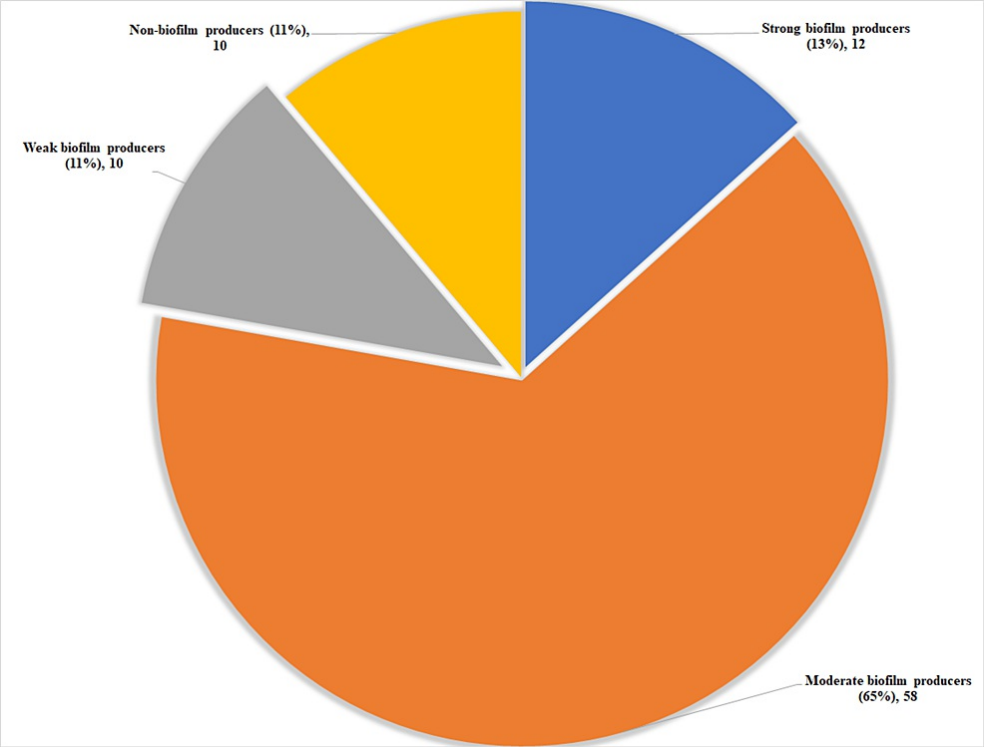
Distribution of enterococcal isolates producing biofilm Quantitation of biofilm production among enterococci isolates is depicted. Numbers indicate the number and percentage of strains that produce biofilm classified as strong, moderate, weak, and non-biofilm producers.

Quantification of biofilm production was estimated for the isolated strains. The OD lower than the cut-off OD (average of all the ODs of the negative control) is considered a non-biofilm producer. OD value less than twice the cut-off OD is considered a weak biofilm producer, OD less than four times of cut-off OD is considered a moderate biofilm producer, and more than four times of cut-off OD is considered a strong biofilm producer. The majority of the strains belonged to moderate biofilm producers followed by strong biofilm producers (Figure 3).

## Discussion

*Enterococcus *is one of the important emerging nosocomial pathogens that causes serious infections, especially among immunocompromised individuals. *E. faecalis* is more resistant than *E. faecium*. So antibiotic susceptibility testing is very important to detect drug resistance. The rate of vancomycin-resistant *Enterococcus* has significantly increased due to extensive use of antibiotics in common and frequent infections such as UTI and GI tract infections and this has a positive correlation with biofilm production.

In the present study, out of 90 *Enterococcus *strains identified, 63 were identified as *E. faecalis*, 25 were identified as *E. faecium* and 2 were identified as *E. gallinarum* which is similar to that of a study from the nearby state. The clinical specimens such as urine, blood, and pus yielded a higher isolation rate compared to other samples. The isolation rate of *Enterococcus *species was higher from samples such as mid-stream urine, blood, and wound aspirates, trailed by wound swabs and body fluids as shown in Table 1. In most of the studies, Enterococci are isolated predominantly from either urine or pus/wound swabs [10,11]. The presence of multidrug-resistant Enterococci isolated from the patient samples is a serious problem as it reduces the treatment options available for the patients. In this study, *E. faecalis* isolated were resistant to penicillin, ampicillin, high-level gentamicin, and ciprofloxacin which is very much in consensus. Meanwhile, the resistance of *E. faecium* was observed to be ceftriaxone along with penicillin, ampicillin, ciprofloxacin, and high-level gentamicin (Table 2). However, in this study, all *Enterococcus *strains were sensitive to linezolid and vancomycin, which is discordant with our study. This difference could be due to the emerging resistance to second-line drugs [12].

Bacterial biofilms obtain the adaptive ability to survive harsh conditions and evade host immune responses. This bacterial property is a menace in hospital settings where nosocomial infections due to biofilm producers increase morbidity and mortality significantly [7]. These are mainly associated with invasive devices and ultimately hamper the therapeutic utility of antibiotics as a last resort. The adaptive response due to their ability to survive against antibiotic therapies, biofilm-specific therapies are warranted, targeting quorum sensing and biofilm maturation process. In this present study, enterococcal strains showed biofilm-producing capacity, that included *E. faecium* and *E. faecalis* but not *E. gallinarum*. Studies showed 5% of isolates to be strong biofilm formers and 78% to be non-biofilm producers which is different from our study. From our observations, biofilm-forming isolates are showing higher resistance to the drugs [13]. Antibiotic resistance patterns will vary from institution to institution or from region to region. Therefore, it is imperative to understand the antimicrobial resistance patterns of the enterococcal isolates to formulate policy guidelines and recommendations for the treatment of *Enterococcus*-associated infections. Furthermore, virulent genes responsible for biofilm production need to be studied to elucidate the relationship between biofilm production and antimicrobial resistance [7,14,15]. *Enterococcus *species inhabit the gut microbiota of the human system and frequently cause biofilm-associated infections, especially among patients admitted to intensive care units and among those who are immunocompromised [16-20]. Catheter-associated UTIs, wound infections, infective endocarditis, and other infections are frequently associated with *Enterococcus *biofilms [7]. Their intrinsic and acquired antimicrobial resistance to different antibiotics and the development of biofilms together limit appropriate clinical management.

The study has limitations of the limited number of samples and lack of follow-up on the patient's clinical management. The samples were selected by random sampling and do not represent the true prevalence in the community. However, the study represents the incidence of *Enterococcus *infections among others in the community and the antimicrobial resistance pattern revealed highly resistant to commonly used antimicrobials and warrants the need to employ appropriate strategies to act against *Enterococcus *strains.

## Conclusions

The emergence and gradual increase of *Enterococcus *with alarming rates of resistance to penicillin, high-level gentamycin, and even emerging resistance to vancomycin pose a serious threat to patient care. This highlights the need for improved surveillance for antimicrobial resistance for enterococci strains emerging from the health care centers and the implementation of antimicrobial stewardship programs. This will enable the rational use of antibiotics and prevent the spread of multidrug-resistant enterococci strains posing an environmental risk.
